# The effects of usual Care in Psychosocial Intervention Trials of patients with coronary artery disease: a systematic review

**DOI:** 10.1186/s13030-020-00180-0

**Published:** 2020-05-12

**Authors:** Hans-Christian Deter, Kristina Orth-Gomér

**Affiliations:** 1 Medical Clinic, Psychosomatics, Charité Universitätsmedizin Berlin, Campus Benjamin Franklin, Hindenburgdamm, 30 12200 Berlin, Germany; 2 Clinical Neuroscience, Karolinska Institutet, Stockholm, Sweden

**Keywords:** Usual care, Coronary artery disease, Psychotherapy, Randomized controlled trials, Review

## Abstract

**Background:**

Many intervention studies of coronary artery disease (CAD) have found health benefits for patients in the “treatment as usual” (TAU) group like in the specific psychotherapy group. In this pilot study, we wanted to examine and discuss the role and reasons for TAU effects.

**Methods:**

By means of a systematic review, we examined the control conditions from psychotherapeutic RCTs with CAD patients related to depressive symptoms, mortality and recurrence rate of events. The review question was limited to factors influencing the TAU effectiveness in such psychotherapeutic outcome studies.

**Results:**

We found a decrease in depressive symptoms in TAU patients (mean ES: 0.65) and very differing mortality and recurrence rates of events. The effects were dependant on the year the study was published (1986–2016), the follow-up time of the study (0.25–7.8 years) and the treatment arms. A small dose of additional counselling, medical attention, and teaching of therapeutic techniques with clinical competence may reinforce the therapeutic alliance. These factors would be possible moderators of control group efficacy related to the reduction in depressive symptoms and a decrease in mortality and events.

**Conclusion:**

In the reviewed studies, we found that the control condition was beneficial for CAD patients, but this benefit was highly variable. Specified psychotherapeutic interventions showed an additional independent effect of treatment on depression and effects on morbidity and mortality. There is a need to identify patients at risk of remaining depressed or under severe stress during usual care. These patients may require additional psychosocial intervention.

## Background

Treatment as usual in coronary artery disease (CAD) is beneficial. Cardiological diagnostic methods and therapeutic options are continuously improving both the prognosis and cardiac health status of patients with coronary artery disease (CAD). However, although CAD is decreasing in many countries, it is still the number one cause of death in patients with heart disease.

Psychosocial trials in cardiovascular prevention and rehabilitation have shown impressive treatment results [1], but there have been further studies that found no differences between active treatment groups and controls. In a recently published meta-analysis [2] with 148 cardiac rehabilitation RCTs, different core components of treatment (patient education (PE; [3, 4]), risk factor modification (RFM; [5, 6]), psychosocial management (PM; [7, 8]), exercise training (ET; [9]), and nutritional counselling (NC; [10]) were associated with reductions in mortality (PM, RFM, ET) and morbidity (PM, PE, ET); all of these core components interacted synergistically to reduce re-vascularization [2]. Psychosocial management aimed at stress management, depressive symptoms and behaviour was an important factor in this analysis.

In the following review, we want to focus on the role and reasons for psychological and physical effects of treatment as usual (TAU), which in general is effective in traditional treatments for CAD. It is applied regularly as a control condition in psychosocial intervention studies. To enable a more comprehensive analysis of physicians medical management it is possible to use physical and psychological data in these studies. Courses of TAU groups were collected in both, CAD trials with positive and negative outcome for the psychotherapy group to examine a broader scope of care and health management activities. However, there is very little research on care-as-usual and even less research on moderators. The problem with moderators is that they are very difficult to examine because all of the trials have too little power to examine them; trials are powered to find effects, not moderators. One solution to solve this problem is to use “individual patient data” meta-analyses to collect the primary data of trials. However, a meta-analysis of this type was not conducted for care as usual conditions until now [11]. Therefore the following review does not examine the various elements of TAU in a systematic way. It is an attempt to evaluate conditions of TAU in major psychotherapeutic treatment trials of CAD and describe in a first step possible mechanisms of efficacy.

In RCTs, “spontaneous remission” of symptoms in the control group is often observed, and in meta-analyses, TAU has been associated with impressive effects in decreasing depressive and physical symptoms [12, 13]. It was concluded that the effect of the psychotherapeutic intervention to be tested was less superior to the control condition: “no evidence that psychological treatments had an effect on total mortality, the risk of re-vascularisation procedures, or on the rate of non-fatal MI, although the rate of cardiac mortality was reduced and psychological symptoms (depression, anxiety, or stress) were alleviated” [14, 15]. Whether the psychosocial intervention provide added benefit compared to TAU alone seems to be one core question. But it is also interesting, whether TAU is inferior or equal to a psychosocial intervention. It depends also on the intensity and quality of TAU and if a low dose of behavioural treatment could be applied (enhanced or intensified TAU). The success in TAU seems to be related to usual medical care conditions, which would also be used in collaborative care [16] or centralized, stepped, patient preference-based treatment studies [17]. In our view, the effect of TAU opens a window to the understanding of different mechanisms. When these mechanisms are optimized, treatment outcomes in CAD health care improves. Both theoretical (a) and practical factors (b) can lead to better TAU effects:

a. An active control group in a psychosocial intervention trial is more powerful than the administration of blinded placebo pills. It is difficult to develop a psychological placebo intervention in a control group, which promises the decrease of psychological symptoms without a psychological treatment component. Transfer and understanding of a credible rationale of a study is part of the intervention [18], and expectations may stimulate a strong placebo effect in the control group compared to the psychosocial intervention itself.

*b. TAU* in CAD represents different duties for the responsible physician in cardiology or primary care [15]. The physician has the key role initiating special treatment options:

- PE, explaining individual diagnostic and treatment strategies to the patient
- strengthening patient’s adherence to drug intake and disease-oriented behaviour counselling about nutrition and other risk factors (RFM)
- treating negative affectivity (PE) and giving advice for ET

The cardiologist with clinical experience and extensive training mediates the benefit and takes the main responsibility in “treatment as usual”. Related to the recent psychosocial literature, a question arises regarding whether the cardiologist should only treat patients physically or whether the cardiologist should also consider psychological and sociological aspects of disease [19, 20]. New scientific results have revealed a novel situation in internal medicine and new challenges for physicians’ behaviour related to psychological concepts [21, 22] and psychotherapeutic treatments [23–26]. The way the physician may approach those bio-psychosocial factors is proposed by the guidelines of cardiological societies that try to provide support for an adequate and differentiated behavioural treatment of cardiovascular disease [27, 28]. However, for physicians of internal medicine, it is uncommon to obtain special psychotherapeutic training. Knowledge and skills are normally acquired in “learning by doing”. Standardized methods were normally not used.

Enhanced, intensified or optimized TAU is a new model for the treatment of physical disease in internal medicine and cardiology.

We wanted to examine the hypothesis, whether additional, clinical and psychosocial activities in TAU have an effect on outcome of depressive symptoms, recurrence of MI, and survival.

## Methods

### Study sample

We examined CAD patients after an acute event - myocardial infarction or unstable angina - defined by angiography in RCTs. Studies were included if they reported a randomized controlled trial of non-pharmacological psychotherapeutic intervention, administered by experienced and trained physicians, psychologists or nurses for adults of all ages with CAD.

### TAU definition

Treatment as usual (TAU) means standard health care of a patient in a region or country offered by physicians, nurses, other health care professionals or hospitals; their costs were covered by public or private insurances. TAU included theoretically some kind of psychological intervention such as counselling, but the criteria for TAU and usual psychological care have rarely been specified.

Additional standardized care activities in the TAU group, e.g. attentional control (TAU plus education), or other interventions were collected.

### Inclusion criteria

To examine TAU mechanisms we focused on very large outpatient studies, with a high sample size (TAU: *N* > 100) and limited treatment targets to obtain comparable conditions. Variability of known and unknown influencing factors will be reduced in this pilot study of TAU.

- non-selected CAD population or CAD population with clinically established psychological disorder.

- cardiac risk factor education as part of intervention.

- psychological intervention also targeted behaviour change for cardiac risk factors.

- treatment targets on depression or type A behaviour.

- individual or group intervention focused on change of thoughts, affects and behaviour.

- treatment components (relaxation, stress management techniques, cognitive techniques, emotional support or client-led discussion, adjunct pharmacology).

### Exclusion criteria

 - trials examining exercise and other core components of cardiac rehabilitation [1].

- specified counselling or multimodal rehabilitation programs with therapeutic aims other than psychotherapy 

- systematic angioplasty.

### Electronic search strategy

**Fig. 1:**
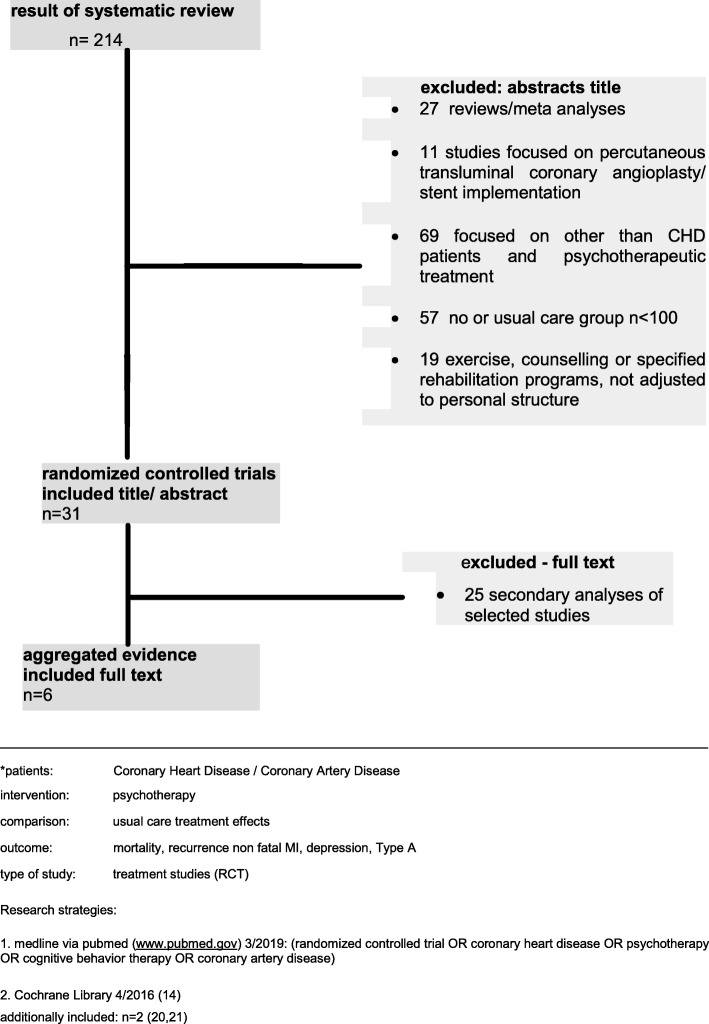
Study flow diagram*

For this review, we used pub med - key words: coronary heart disease; coronary artery disease; psychotherapy; cognitive behavioural therapy; randomized controlled trial. Searches of multiple electronic databases up to March 2019 were conducted, supplemented by hand-searching of identified reviews and citation tracing of eligible studies. From this review we selected out of 214 six studies (Fig.1).

Additional two studies from the updated Cochrane review [14] were selected.

The Cochrane review until April 2016, included thirty five trials with 10,703 patients. Of these, ten studies examined psychological interventions targeted at people with a confirmed psychopathology at baseline. 11 trials recruited people with varying levels of psychopathology, three studies excluded people with psychological conditions, and 11 studies did not report psychological status. Due to our selection criteria to examine usual care conditions in psychotherapeutic trials 3 out of 35 in the Cochrane review included studies and 3 there not included studies were used in the following analysis.

In the selected studies from this systematic reviews we compared the TAU arm samples (> 100 patients in each group) according to the outcome of psychological symptoms (depression, type A behaviour), recurrent events and mortality. The scope of this review question was limited to important factors influencing the effectiveness of TAU in the selected psychotherapeutic outcome studies.

### Statistical analysis

In this review, TAU groups of selected studies were described related to chosen clinical, cardiological and psychological factors. The statistical analyses were performed with calculation of effect size (Cohens d) for detecting differences in depression and type A outcome of individual TAU-groups. Mortality and recurrent MI were presented in percentage of events between baseline and follow up of studies.

## Results

We reviewed the control condition (TAU) in eight RCTs (3998 patients), which differed substantially in the selected TAU samples (Table 1). The outpatient psychotherapeutic intervention studies published between 1986 and 2016 focused on depression or stress management and showed psychological or clinical effects in CAD patients "[19–26]. Many of these intervention studies in Europe could demonstrate a health benefit [29], but the usual care group in RCTs also exhibited a remission of symptoms [19–22]. High-risk behaviour [19], such as insufficient adherence with internal treatment regime/ prescriptions [23], high psychosocial stress/strain exposure, psychic symptoms or psychiatric co-morbidities [23–25], was measured.

**Table 1:** Specific characteristics examining 3998 patients in the TAU arm of eight reviewed psychotherapeutic RCTs on coronary artery disease

Randomized Controlled Trial Year of publication;	No. of patients in TAU, % male	Age mean sd	% heart failure	Psychological selection	Effect on study aims in TAU: -all cause mortality rate/year -recurr. Non-fatal MI/y.
RCPP [19] 1986	*N* = 270 90% male	53.6 ± 6.1	10.2% congest. Heart failure	no	mortality/y: 1.6% rec. non-fatal MI/y: 3.8%
Jones and West [20] 1996	*N* = 1079 gender not specified	“all ages with few practical exclusions“	21% “disability”	no	mortality/y: 7.2% recurrent non-fatal MI/y: 5%
M-HEART [21] 1997	*N* = 684 65% male	59.3 ± 11.1	LVEF ≤35%	GHQ ≥ 5 16%;	mortality/y: 5.4%
ENRICHD [22] 2003	*N* = 1243 56% male	61 ± 12.5	25% severe dysfunction of EF	Major, minor depressed	mortality/y: 5.7% rec. non-fatal MI/y: 5.7%
CREATE [23] 2007;	*N* = 142 75% male	57.3 ± 8.4	CCS 4 excluded	Moderate or severe depressed	mortality /3 mo: 0 non-fatal MI/3 mo: 1.5%
SWICHD [24] 2009;	*N* = 125 100% female	62.7 ± 8.7	17.9% EF ≤ 40%	no	mortality/y: 2.8%
SUPRIM [25] 2011	*N* = 170 75% male	61.0 ± 8.28	26.8% heart failure	no	mortality/y: 1.9% rec. non-fatal MI/y: 3.8%
SPIRR-CAD [26] 2016	*N* = 285 79% male	59.3 ± 9.3	NYHA III-IV 15.1%	mild to moderate depression	mortality/y: 1.6% MACE/y: 4.2%

We found a decrease in depressive symptoms in up to 48% of the control cases. The mortality and recurrence rate of events was broadly associated with the year of publication (1986–2011) with the highest mortality in studies published from 1996 to 2003 [20–22], the follow up time of the study (0.25–7.8 years; more than 4.5 years: [19, 24, 25]) as well as the severity of cardiac (heart failure: 10–25%) and psychiatric disease (mild to high severity of depressive symptoms: [20–22, 26]; no selection related to psychiatric disease: [19, 20, 24, 25]); health care outcome was equal to better health outcome. Several aspects of the TAU-follow up results can be specified and the magnitude of change in usual care estimated, whether additional, clinical and psycho social activities in TAU have an effect on outcome of survival, recurrence of MI, and depressive symptoms.

### Comparison of mortality and MI-recurrence rate per year in examined studies

a. The mortality per year fluctuated between 1.6% [19, 26] and 7.2% [20]. Age of patients, gender, percentage of heart failure, and severity of psychological symptoms could be responsible for these variation (Table 1).

Considering the survival rate at the end of the individual study and in projection of these data to an estimated follow-up interval of 8 years in order to compare survival in all presented TAU groups, we found impressive differences with advantages for RCPP [19], SUPRIM [25] and SPIRR-CAD (26; Table 2). An old study with a younger patient group [19], a new study [26] and a study with a long follow-up time [25] demonstrated the highest survival rates (84.8 to 87.2%).

**Table 2:** Treatment as usual (TAU) differs in eight psychotherapeutic RCTs on coronary artery disease and outcome related to psychological symptoms and survival

	Enhanced TAU			Outcome in TAU		
Randomized Controlled Trial	Years in the study	No of personal examinations	Additional therapeutic activity	Reduction of psychological symptoms	Survival rate (%) at end of study	Estimated survival rate within 8 years^1^
RCPP [19]	4.5 y.	5 (3 cardiologic, 2 psycho logical)	33 group sessions à 90 min.	Type A behaviour: −9.8%; ES^2^: 0.72; 95%CI 0.89–0.56	92.8%	87.2
Jones and West [20]	1 y.	3	0	depression not specified for TAU	92.8%	42.4%^1^
M-HEART [21]	1 y.	3 (1 cardiological, 3 psychological)	0	BDI depression: - 9.5%; ES^2^: 0.1; 95%CI 0.26–0.07	94.6%	56.8%^1^
ENRICHD [22]	2.4 y.	5 (cardiolog.,psychological)	Active partnership Health booklet	BDI depression: −33%; ES^2^: 0.69; 95%CI 0.85–0.53	86.2%.	54.3%
CREATE [23]	0.25 y	2 (cardiolog.,psychological)	12 × 20 min clinical management	BDI: − 40.3% HAMD:-48%; ES^2^: 1.81; 95%CI 2.2–1.42	100% /	?
SWICHD [24]	7.1 y.	1(cardiolog, psychological; 2x questionnaires by letter)	0	depressive symptoms: −16.1%; ES^3^: 0.25; 95%CI 0.64–0.14	80.1% /	77.6%
SUPRIM [25]	7.8 y.	5 (cardiological, psychological)	0	Depression not reported	85.2% /	84.8%
SPIRR-CAD [26]	2 y.	5 (2x cardiologic, 5x psychological)	30 min information on risk factors	HADS-D − 13.4%; ES^4^:0.41 95%CI 0.65–0.18 remission on HADS-D 35.8%	96.8% /	87.2%

^1^ 8 years survival rate was predicted using survival data of the individual study. Presented studies differ in time

between 0,25 years and 7.8 years. Mortality is higher in the year after the event. So in studies with a one or two

years follow up period the estimated value of 8 years survival may be higher.

^2^mean and standard deviation at baseline and follow up (RCCP:4.5 y.;M-Heart:1 y.; ENRICHD (depressed

participants only): 6 months; CREATE:3 months)

^3^ mean and standard deviation at baseline and follow up (1–2 years), published by Koertge J et al. J Intern Med

2007;263:281–293

^4^LOCF ANCOVA mean and standard deviation at baseline and follow up (24 months)

b. Recurrent non-fatal MI per year we found between 3.8% [19, 26] and 5.7% [22]. RCPP [19] had the lowest mean age and was like SUPRIM [25] not selected related to psychological symptoms. ENRICHD [22] included a high percentage of women and older patients with depressive symptoms.

Different cardiological and emergency care within 30 years, the different follow up time, the severity of cardiac or psychiatric disease seems not to explain all these differences. TAU is provided by cardiologists and GPs, and mortality and recurrent non-fatal MI may decrease with improvement of the medical management in post-MI patients in the examined countries.

Additional cardiological or behavioural treatment may have an influence on events and mortality. *Possibly moderators* of this outcome in TAU can be identified [1, 14]:

1. A well organized cardiological treatment program for the treatment and the TAU group within a RCT [19, 25] seem to be beneficial.
2. High impact intervention with a high number of personal clinical and psychological examinations during the study [19, 25, 26] may have therapeutic effects related to adherence, risk factor management, and the psychological adaptation of patients.
3. Adverse effects in control conditions: In some studies recurrence of MI [19, 25] or mortality [19, 24] was higher in the TAU arm. Differences to be related to the behavioural treatment arm are shown and demonstrate limitations of the TAU effect.

### Comparison of psychological symptom change (ES) in examined studies

Additional behavioural or cardiological treatment may influence course of psychological symptoms in TAU, which showed in selected studies low (0.1 [21]) and high ES (1.81 [23]):

1. The different applied psychotherapeutic treatment components (treating: by physicians or psychologists type A [19], depressive symptoms [22, 23, 26] or by nurses [21] or stress management by psychologists [20] or trained nurses [24, 25]) in the treatment arms of reviewed studies seem to have no influence on TAU effect.
2. Some studies present an additional dose of special counselling in the TAU arm (30 min [26]), a medium dose (12 × 20 min [23].) or a high dose (33 × 90 min [19]), which seems to be beneficial in TAU.
3. In addition to these study differences, which may influence control group efficacy [30] the effect in this group seems also to depend on the quality of care. - This care is mostly provided by the physician with his clinical competence, applied techniques, and the therapeutic methods. Especially his clinical skills, behaviour and ability to create a helpful alliance may be important for the outcome in the TAU arm.

Data of one psychotherapeutic outcome study [26] demonstrated the efficacy of different levels of care. CAD patients with mild to moderate depression were treated in a stepped care setting by the following methods: 1. by TAU with cardiologists or GP, 2) by three additional hours of psychodynamic or systemic psychotherapy, and 3) by 25 additional sessions (90 min.) of group psychotherapy using psychodynamic and behavioural strategies. The TAU group received a 30 min individual session to inform the patients about their disease, and all patients received 5 physical and/or psychological examinations during the study.

The result of this study is remarkable: all three therapeutic settings were approximately equally helpful, and depressive symptoms decreased significantly in all groups [26]. It seems that the personal and cardiological care for the patients in group 1 was sufficient, without the need for further psychotherapeutic or psycho-pharmacologic interventions. However, the subgroup of depressive patients with negative affectivity and social inhibition (type D) reduced depressive symptoms much further in the psychotherapy arm than in the TAU arm [26].

So the question emerges, whether CAD patient needs usual, augmented or specialized care.

## Discussion

In a systematic review we wanted to focus on the role and reasons for psychological and physical effects of treatment as usual (TAU), which in general are effective in traditional treatments for CAD. For that we examined eight major RCT’s of CAD patients after myocardial infarction or unstable angina defined by angiography. We focused in these psychotherapeutic studies on outcomes of usual care arms. TAU was applied regularly as a control condition in these intervention studies. In a more comprehensive analysis of physicians medical management it was possible to use physical and psychological data in these studies. Studies 19 and 24 showed reduced mortality rates, studies 19 and 25 showed reduced morbidity of CAD in the psychotherapeutic treatment compared to TAU. But between studies we found differences in the TAU groups, which not depended from year the study was done or the years of follow-up: A beneficial cardiological treatment program for the treatment within a RCT [19, 25] seem to be a moderator/mechanism for a beneficial cardiological development. Additionally a great number of personal clinical and psychological examinations during the study [19, 22, 25, 26] and additional a limited therapeutic activity [19, 23, 26] had presumably good psychological TAU effects.

In summary, the results of the examined psychotherapeutic studies were positive for patients’ health: Studies 21 to 26 showed a reduction in depressive symptoms. Although it was never specifically expressed, the data indicate health benefits for patients participating in any study group. As moderators of TAU efficacy a great number of clinical and psychological examinations during the study, an additional dose of special counselling, clinical disease management or group sessions in some studies seemed to be beneficial for decrease of depressive symptoms or type A personality. In addition to these study differences, which may influence control group efficacy [30] we hypothesize the effect in this group seems also to depend on the quality of care, which could not be examined in this review. But we believe the following *hypothetical mechanisms* could be important:

*TAU efficacy related to cardiological outcome.*

The research team has more time and interest to devote to the study of patients in any clinical situation. So study patients have *better “usual care” conditions* than patients in standard care.

*TAU efficacy related to depressive symptoms.*

1. A “*Spontaneous remission*” may illustrate the course of disease after a cardiac event. The course of TAU also covers interactions generated by the patient himself and by care professionals. A remission could be evoked by increased hope and stronger resistance of an individual. Patients who are depressed and/or compensate for their feelings could react more actively.
2. Patients obtained higher medical and social support from their cardiologists during the clinical intervention before randomization [19–26]. This profound experience can lead to the argument that the reduction in depressive symptoms in the TAU arm is triggered by a kind of “*positive expectation*” or “placebo response” [22, 23].
3. *TAU intensity:* What therapeutic dose of TAU is necessary in cardiac patients to reduce depressive symptoms? We maintain that the control status and the control group individually differ between selected studies: In the SPIRR-CAD trial one additional information session (30 min.) and five study examinations within 18 months are sufficient to decrease depressive symptoms in a sub group (35.8%) of all patients [26]. In the ENRICHD trial almost half of the control participants (who received usual care plus education) also received anti-depressant medication, psychotherapy and participated in cardiac rehabilitation. It is not surprising that they showed improved symptoms of depression—albeit less that the group that received the psychological intervention [22].
4. *TAU quality:* Regarding the therapeutic quality of TAU, was it possible for the physician to give the symptom a name, or a diagnosis, which was in line with the subjective theory of the patient and convinced him, inspired confidence and positive expectation [31]? Did the physician take the patient seriously, did he inform the patient about the next diagnostic or therapeutic steps and the procedure of therapy? Had he assured himself that the patient cooperated?

However, we could not examine how the well-known mediators of treatment outcome in psychotherapeutic trials of depression, e.g., therapeutic alliance [32], expressed empathy or dysfunctional thinking [33], in individual physician-patient contacts in the selected studies were effective.

We do not know in which way these mechanisms may influence adherence to medication, or progress of coronary disease. It seems necessary to examine and understand all effective therapeutic mechanisms which give support for enhanced cardiological and psychological usual care in CAD patients. This knowledge should present as a basic condition for additional behavioural therapeutic activities.

### Limitations

We reviewed the control conditions of psychotherapeutic intervention trials related to outcome of psychological and physical symptoms. In this pilot analysis of usual care outcome during a psychotherapeutic trial different mechanisms could be demonstrated. Other usual care effects may be missed:

- Varying mortality and recurrence rates are difficult to assess without comparison - notably patients with CAD who did not volunteer for trials. There may be a selection bias, patients participated in the trials who had higher treatment adherence than not included patients.

- Comparisons of usual care in studies with smaller sample size or comparisons to surgery, exercise training, counselling, or multimodal rehabilitation may show different results.

- We had no information if additional treatment activities on a lower level were integrated in TAU: e.g. patient education [3], risk factor management [4–6], psychosocial modification [7, 8, 19, 21–26], nutrition counselling [10], or exercise training [9].

In our review we have integrated the effect size, but due to some difficulties in performing such analyses we have not evaluated the heterogeneity, and not performed subgroup analyses by the method of meta-analysis. Such quantitative analyses and subgroup analyses would have increased the validity of the study and revealed the strategy to optimize the treatment.

In this first review of usual care samples from major CAD outcome studies, all these data are missing. These data would detect further predictors of usual care effects. However, it is difficult to examine all these interactions in one review [15].

In summary, the results in this review were not unexpected. A variety of factors have been cited to explain improvements in the TAU arms in depressive symptoms including regression to the mean, spontaneous remission, non-specific effects of attention, and concurrent therapies [30].

However, the cardiologist is responsible for the physical condition and prognosis of his patients [19, 25, 27]. According to the Cardiovascular Guidelines [28] the physician must select the optimum TAU or special psychosocial intervention that is appropriate to diminish depressive symptoms or risk behaviour in a patient [34].

## Conclusion

We reviewed the control conditions of psychotherapeutic interventions in general terms. If the results related to probable mechanism of cardiological and psychological outcome can be repeated, confirmed and widely distributed, the positive effects on cardiological standard care, population health and costs [35] would be considerable. Based on the result of this pilot study, the following treatment strategy should be verified in further studies: “The responsible cardiologist or GP allowed additional time for counselling and modifying of risk factors, for providing psychosocial management, and for giving advice for exercise training [9] and patients will experience a health benefit”. Therefore, it is necessary to identify mechanism which predict health outcome in patients at risk, either to remain depressed or to remain under severe stress, and patients who have no such risk and do not need additional psychosocial intervention.

## Data Availability

Not applicable.

## Abbreviations

CAD: Coronary artery disease
CHD: Coronary heart disease
CREATE: Canadian cardiac randomized evaluation of antidepressant and psychotherapy efficacy
ES: Effect size
ET: Exercise training
ENRICHD: Enhancing recovery in coronary heart disease patients
GP: General practitioner
M-HEART: Montreal heart attack readjustment trial
NC: Nutritional counselling
PE: Patient education
PM: Psychosocial management
RCPP: Recurrent coronary prevention project
RCT: Randomized controlled trial
RFM: Risk factor modification
SPIRR-CAD: Stepwise psychotherapy intervention for reducing risk in coronary artery disease
SUPRIM: Secondary prevention in uppsala primary health care project
SWICHD: Stockholm women’s intervention trial for coronary heart disease
TAU: Treatment as usual
